# MRI-Guided Focused Ultrasound as a New Method of Drug Delivery

**DOI:** 10.1155/2013/616197

**Published:** 2013-05-12

**Authors:** M. Thanou, W. Gedroyc

**Affiliations:** ^1^Institute of Pharmaceutical Science, King's College London, Franklin-Wilkins Building, Stamford Street, London SE1 9NH, UK; ^2^Department of Experimental Medicine, Imperial College, St. Mary's Hospital, Praed Street, London W2 1NY, UK

## Abstract

Ultrasound-mediated drug delivery under the guidance of an imaging modality can improve drug disposition and achieve site-specific drug delivery. The term focal drug delivery has been introduced to describe the focal targeting of drugs in tissues with the help of imaging and focused ultrasound. Focal drug delivery aims to improve the therapeutic profile of drugs by improving their specificity and their permeation in defined areas. Focused-ultrasound- (FUS-) mediated drug delivery has been applied with various molecules to improve their local distribution in tissues. FUS is applied with the aid of microbubbles to enhance the permeability of bioactive molecules across BBB and improve drug distribution in the brain. Recently, FUS has been utilised in combination with MRI-labelled liposomes that respond to temperature increase. This strategy aims to “activate” nanoparticles to release their cargo locally when triggered by hyperthermia induced by FUS. MRI-guided FUS drug delivery provides the opportunity to improve drug bioavailability locally and therefore improve the therapeutic profiles of drugs. This drug delivery strategy can be directly translated to clinic as MRg FUS is a promising clinically therapeutic approach. However, more basic research is required to understand the physiological mechanism of FUS-enhanced drug delivery.

## 1. Introduction

Therapeutic high intensity focused ultrasound (HIFU) or Focused Ultrasound (FUS) is a noninvasive medical treatment that allows the deposition of energy inside the human body. Frequencies of 0.8–3.5 MHz are generally used during the clinical applications of FUS. The energy levels carried in the ultrasound beam are several orders of magnitude greater than those of a standard diagnostic ultrasound beam. In the case of focused ultrasound, the ultrasound waves can be focused at a given point. The high energy levels carried in a HIFU beam can therefore be magnified further and delivered with precision to a small volume, while sparing surrounding tissues. FUS energy can be deposited in small areas providing a substantial advantage for drug targeting. The volume of energy deposition following a single HIFU exposure is small and will vary according to transducer characteristics but is typically cigar shaped with dimensions in the order of 1–3 mm (transverse) 8–15 mm (along beam axis) [1]. HIFU transducers deliver ultrasound with intensities in the range of 100–10,000 W/cm^2^ to the focal region, with peak compression pressures of up to 30 MPa peak and rarefaction pressures up to 10 MPa [2]. The ultrasound wave propagates through tissues, causing alternating cycles of increased and reduced pressure (compression and rarefaction, resp.). In the case of tissue ablation during HIFU treatments, the temperature at the focus can rise rapidly (up to 80°C) which can cause cell damage. “Inertial cavitation” occurs simultaneously with tissue heating. Ultrasound affects the molecular structure of the tissues during the alternating cycles of compression and rarefaction. During rarefaction, gas can be drawn out of the solution to form bubbles, which can collapse rapidly. In this case injury is induced through a combination of mechanical stresses and thermal effects at a microscopic level. When Ultrasound is applied in biological systems it can induce local tissue heating, cavitation, and radiation force, which can be used to initiate local (focal) drug delivery, increase permeation through membranes, and enhance diffusivity of drugs, respectively, only at the site of sonication therefore allowing control of local drug release [3].

The ability of FUS to induce thermal or mechanical effects at very defined (focal) locations in living tissue has been first described in 1942, when Lynn et al. tested FUS [4] in the brain. In the 1950s Fry brothers developed a clinical FUS device for treating patients with Parkinson disease. They used a sonication system in combination with X-rays to determine the target location relative to skull and to focus the ultrasound beam through a craniotomy into deep brain for effective functional neurosurgery [5]. Later on, in the 1980s the first FDA-approved FUS system, Sonocare CST-100, was developed to treat ocular disorders such as glaucoma and many patients were successfully treated with this system [6]. More recently substantial technological developments have led to new FUS equipment for a number of different applications. Current research and development aims to explore transducer technology and array design to achieve faster delivery of focal sonications, to improve transducer accessibility (smaller devices) or fit them to certain parts of the body such as a helmet of arrays for brain focal treatment.

Several FUS devices are investigated currently in clinical trials. These devices can operate under image guidance to provide real-time monitoring of the treatment.

Guidance and monitoring of acoustic therapy controls the treatment region and minimizes damage to adjacent structures. Monitoring using real-time imaging, such as with sonography (diagnostic ultrasound), ensures that the targeting of the FUS beam is maintained on the correct area throughout the procedure. MRI and sonography are the two imaging modalities currently being used for guidance and monitoring FUS therapy. MRI has the advantage of providing temperature data during FUS treatment. However, MRI guidance is expensive, labor-intensive, and of lower spatial resolution in some cases. Sonographic (ultrasound) guidance provides the benefit of imaging using the same form of energy that is being used for therapy. The advantage of this is that the acoustic window can be verified with sonography. Therefore, if the target cannot be well visualised with sonography, then it is unlikely that FUS therapy will be effective. Temperature monitoring using sonography is not yet available [2]. InSightec manufactures the ExAblate2000 which uses MRI for extracorporeal treatment of uterine fibroids (FDA-approved) with significant success, and extensive current research focuses on investigating its application in other parts of the body [7, 8]. ExAblate technologies are used for prostate cancer or bone metastasis (ExAblate 2100 Conformal Bone System); these applications are currently under development by InSightec. The Ablatherm HIFU/US consists of a transrectal probe for prostate treatment and has CE mark approval [9]. The Sonablate 500, an ultrasound guided system uses a transrectal probe to carry out prostate cancer focal ablation [10]. The Sonalleve HIFU/MR is an MR compatible device developed to examine a series of applications as fibroids and other body sites [11]. A recently introduced device is the transcranial MR-guided focused ultrasound. This is a hemispheric phased-array transducer (ExAblate Neuro; InSightec Ltd., Tirat Carmel, Israel) with each element driven separately, providing individual correction of skull distortion as well as electronic steering. The device received CE Mark for neurological disorders recently (December 2012). The device has been used for the treatment of neuropathic pain essential tremor and there is also evidence of possible application for brain tumours [12, 13]. Essential tremor noninterventional functional neurosurgery treatment has shown an immense potential of transcranial MRgFUS application to induce lesions focally and treat patients nonsurgically [14]. 

## 2. Fundamentals of Focused Ultrasound Treatments

Ultrasound propagates as mechanical vibrations that induce molecules within the medium to oscillate around their positions in the direction of the wave propagation. The molecules form compressions and rarefactions that propagate the wave. The ultrasound energy is decreased exponentially through the tissue. The decrease in acoustic energy per unit distance travelled is called “attenuation.” The rate of energy flow through a unit area, normal to the direction of the wave propagation, is called acoustic intensity. At 1 MHz the ultrasound wave is attenuated about 50% while it propagates through 7 cm of tissue. The attenuated energy is transformed into temperature elevation in the tissue [15, 16].

Ultrasound is transmitted from one soft tissue layer to another. Usually in soft tissues a small amount of wave is reflected back except at the soft tissue-bone interface where approximately one-third of the incident energy is reflected back. In addition, the amplitude attenuation coefficient of ultrasound is about 10–20 times higher in bone than in soft tissues. This causes the transmitted beam to be absorbed rapidly within the bone [17]. Ultrasound induces mechanical vibration of the particles or molecules of a material. Each particle moves small distances from its rest position but the vibrational energy is propagated as a wave traveling from particle to particle through the material. Ultrasound is attenuated as it travels through a tissue due to beam divergence, absorption, and deflection of the acoustic energy. Deflection consists of the processes of reflection, refraction, and scattering. The energy required for a sound wave to travel through a tissue must overcome the internal friction intrinsic to any material. As a sound wave travels through tissue, it continually loses a proportion of its energy to the tissue (attenuation). The reasons of attenuation are divergence, deflection, and absorption. Divergence of the sound beam spreads the acoustic energy over a larger beam area and reduces the intensity along the beam axis. Deflection of acoustic energy out of the beam also reduces the intensity. The greatest cause of attenuation in the body is absorption, in which energy is transferred from the sound beam to the tissue and ultimately is degraded to heat. The amount of absorption depends on the frequency of the ultrasound beam. Whenever a sound beam encounters a boundary between two materials, some of the energy is reflected and the remainder is transmitted through the boundary. The direction of the reflected wave, or the echo, depends on the orientation of the boundary surface to the sound wave. The major physical effects of ultrasound are heat, mechanical effects, cavitation, and chemical effects. Acoustic impedance is a measure of the resistance that a material offers to the passage of an ultrasound wave and is expressed in units of rayls (kg/m^2^/sec). Acoustic impedance of water is 1.5 × 10^−6^ Mrayls whereas that of bone is 8 × 10^−6^ Mrayls. The greater the difference in acoustic impedance between two materials, the stronger the echo (reflected wave) arising from their interface. Heat is the most common physical effect generated by sound waves in the body. When the rate of heat generation is higher than the rate of heat dissipation in the body, the body temperature will rise significantly. Temperatures above 43°C if maintained for extended period can be damaging. Mechanical effects, such as the breaking of bonds, can occur if the amplitude of the ultrasound wave is significantly large. Cavitation occurs when an ultrasound beam of sufficient intensity travels through a liquid in which gas bubbles have been generated. The alternating high- and low-pressure periods of the ultrasound wave forces the bubbles to contract and expand. The amplitude of the bubble oscillation increases with increasing ultrasonic intensity. During the bubble contraction, the internal pressure can increase and the temperature can reach 10,000°C. A sonic explosion can occur, releasing a large amount of energy, although for very short (*μ*m) distances. Tissues and cells in the vicinity can be damaged. Cavitation is the responsible mechanism for the disintegrations of stones in lithotripsy. Chemical effects, such as the acceleration of chemical reactions, can occur due to an increase in the temperature and pressure. These effects would be expected in high-intensity ultrasound fields [18]. When ultrasound beams are focused a focal diameter of 1 mm can be achieved at 1.5 MHz. The length of the focus is 5–20 times larger than the diameter. If the ultrasound beam is transmitted from an applicator 2–3 cm in diameter, the ultrasound intensity at the millimeter-sized focal spot can be several hundred times higher than in the overlying tissues. Typical diagnostic ultrasound transducers deliver ultrasound with time-averaged intensities of approximately 0.1–100 mW/cm^2^ or compression and rarefaction pressures of 0.001–0.003 MPa, depending on the mode of imaging. In contrast, HIFU transducers deliver ultrasound with intensities in the range of 100–10,000 W/cm^2^ to the focal region, with peak compression pressures of up to 30 MPa and peak rarefaction pressures up to 10 MPa [2]. The ultrasound exposure drops off rapidly across the area within the sonication path and therefore focusing provides a method to overcome attenuation losses and to concentrate energy deep in the body while avoiding the surrounding tissues [19].

Focusing is dramatically improved with the use of transducer arrays that are driven with signals having the necessary phase difference to obtain a common focal point. The main advantage of these phased arrays is that the focal spot can be controlled. In addition, the electronically focussed beam allows multiple focal points to be induced simultaneously or fast electronic scanning of the focal spot which increases the size of the focal region. This feature allows shorter treatment time [20, 21].

## 3. Image Guided Focused Ultrasound Mediated Drug Delivery

### 3.1. Using Clinical Imaging and Drug Delivery Systems

The combination of high-intensity focused ultrasound together with high-resolution MR guidance has created a system that can produce tissue destruction deep within solid organs without any invasion. Accurate targeting and detailed accurate thermal mapping are provided by MRI [22].

In recent years imaging has been combined with FUS to provide real-time manipulation of drug guidance within the targeted area. Ultrasound and magnetic resonance (MR) imaging are widely used clinical imaging modalities that can be combined with FUS for image guided FUS treatments. In the area of drug delivery ultrasound microbubbles or nanocarriers providing contrast enhancement can be used.

When using nanocarriers sensitive to mechanical forces (the oscillating ultrasound pressure waves) and/or sensitive to temperature, the content of the nanocarriers can be released locally. Thermosensitive liposomes have been suggested for local drug release in combination with local hyperthermia more than 25 years ago. Microbubbles may be designed specifically to enhance cavitation effects. Real-time imaging methods, such as magnetic resonance, optical and ultrasound imaging, have led to novel insights and methods for ultrasound triggered drug delivery. Image guidance of ultrasound can be used for: (1) target identification and characterization; (2) spatiotemporal guidance of actions to release or activate the drugs and/or permeabilize membranes; (3) evaluation of biodistribution, pharmacokinetics and pharmacodynamics; and (4) physiological read-outs to evaluate the therapeutic efficacy.

### 3.2. FUS Induced Increase in Temperature for Tissue Specific Drug Release from Thermosensitive Carriers

Liposomes show significant advantages for drug delivery in tumours. The enhanced permeability and retention effect has served as a basic rationale for using liposomes and other nanoparticles to treat solid tumors. However, it has been recently noticed that the enhanced permeation and retention effect does not guarantee a uniform delivery. This heterogeneous distribution of therapeutics is a result of physiological barriers presented by the abnormal tumor vasculature and interstitial matrix. In a recent review by Jain and Stylianopoulos, the barriers of tumour nanoparticle delivery were summarised. First, the abnormal structure of tumor vessels results in heterogeneous tumor perfusion and extravasation, and a hostile tumor microenvironment that supports drug resistance and tumor progression. Second, in highly fibrotic tumors, the extracellular matrix blocks penetration of large nanoparticles leaving them concentrated in perivascular region. To overcome these barriers the authors suggest normalization of the vascular network and the extracellular matrix as well as development of nanoparticles that release therapeutic agents in response to the tumor microenvironment or an external stimulus (such as heat light and HIFU) [23].

Thermosensitive carriers have a long presence in research and development. Yatvin et al. first described the effect of hyperthermia on liposomal carriers in 1978 [24]. However, development of thermosensitive liposomal carriers for cancer was only introduced as recently as 1999 when Needham's group evaluated phase transition enhanced liposomal permeability [25]. *In vivo *data using cancer models were presented one year later when the authors described a new lipid formulation containing doxorubicin optimized for mild hyperthermic temperatures (39°C to 40°C) that are readily achievable in the clinic leading to very rapid release times of the drugs. This new liposome, in combination with mild hyperthermia, was found to be significantly more effective than free drug or current liposome formulations at reducing tumour growth in a human squamous cell carcinoma xenograft [26]. These low temperature-sensitive liposomes (LTSL) were further developed in dogs having canine tumours to show a superior efficiency [27, 28]. A formulation based on these thermosensitive liposomes took the brand name Thermodox and was further developed by Celsion corporation. Thermodox liposomes can be triggered to release their payload by any heat-based treatment such as radiofrequency thermal ablation (RFA), microwave hyperthermia, and high intensity focused ultrasound (HIFU). Results from a Phase I study that used Thermodox was recently published [29]. In a Phase I study researchers used escalating dose of Thermodox with radiofrequency (RF) ablation and concluded that Thermodox can be safely administered at 50 mg/m^2^ in combination with RF ablation. Currently Thermodox in combination with RF ablation is being tested in a large Phase I study to treat hepatocellular carcinoma [30]. 

The concept of using liposomes and HIFU was introduced recently, in 2006 when Frenkel et al. used liposomal doxorubicin (Doxil) in combination with pulsed high-intensity focused ultrasound (HIFU) exposures in a murine breast cancer tumor model. Doxil is a stable liposomal preparation that has no response to increased temperature [31] and was developed to minimise doxorubicin's cardiotoxicity, by encapsulating doxorubicin within stealth liposomes. Although Doxil achieves long circulation of doxorubicin with minimum cardiotoxicity it does not rapidly release the drug within the tumour. Pulsed-HIFU exposures were not found to enhance the therapeutic delivery of doxorubicin and did not induce tumour regression. However, a fluorescent dextran showed blood vessels to be dilated as a result of the exposures. Experiments with polystyrene nanoparticles of similar size to the liposomes showed a greater abundance to be present in the treated tumours [32]. Although this study did not achieve or prove a therapeutic advantage of the use of HIFU with temperature stable liposomes it showed clearly that pulsed HIFU induces a substantial increase of permeation of macromolecules and nanoparticles in tumours. 

In 2007 Dromi et al. presented the first study on thermosensitive liposomes (Low Temperature Sensitive Liposomes (LTSL)) and HIFU. The authors investigated pulsed-high intensity focused ultrasound as a source of hyperthermia with thermosensitive liposomes to enhance delivery and efficacy of doxorubicin in murine adenocarcinoma tumours. *In vitro* treatments simulating the pulsed-HIFU thermal dose (42°C for 2 min) triggered release of 50% of doxorubicin from the thermosensitive liposomes; however, no detectable release from the nontemperature sensitive liposomes (similar to Doxil) was observed. Similarly, *in vivo* experiments showed that pulsed-HIFU exposures combined with the LTSL resulted in more rapid delivery of doxorubicin as well as significantly higher concentration within the tumour when compared with LTSLs alone or nonthermosensitive liposomes, with or without exposures [33]. 

In a later study the same team developed MR imageable thermosensitive liposomes (iLTSL), with the objective to characterise drug release in phantoms and *in vivo*. An MRI contrast agent (ProHance^®^ Gd-HP-DO3A) and doxorubicin were loaded and drug release was quantified by spectroscopic and fluorescence techniques, respectively. Release with HIFU under MR guidance was examined in tissue-mimicking phantoms containing iLTSL and in a VX2 rabbit tumour model. iLTSLs demonstrated consistent size and doxorubicin release kinetics. Release of doxorubicin and ProHance^®^ from iLTSL was minimal at 37°C but fast when heated to 41.3°C. Relaxivity of iLTSL increased significantly from 1.95 ± 0.05 to 4.01 ± 0.1 mMs^−1^ when liposomes were heated above the phase transition temperature indicating the release of ProHance^®^ from liposomes and its exposure to the aqueous surroundings. Importantly, the signal increase corresponded spatially and temporally to MR-HIFU-heated locations in phantoms. *In vivo*, the investigators confirmed MRI signal after i.v. iLTSL injection and after each 10-min heating, with greatest increase in the heated tumour region. The authors concluded that MR-HIFU combined with iLTSL may enable real-time monitoring and spatial control of drug release from liposomes [34].

In a follow-up study the authors investigated the effect of iLTSL in rabbits bearing VX2 tumours. In that study image-guided noninvasive hyperthermia was applied for a total of 30 min, completed within 1 h after LTSL infusion and quantified doxorubicin release in tumours with HPLC and fluorescence microscopy. Sonication of VX2 tumours resulted in accurate and spatially homogenous temperature control in the target region. LTSL+MR-HIFU resulted in significantly higher tumour doxorubicin concentrations (3.4-fold greater compared LTSL resp.). The authors observed that free doxorubicin and LTSL treatments appeared to deliver more drug in the tumour periphery as compared to the tumour core indicating that HIFU induced hyperthermia and LTSL increases doxorubicin's permeability as doxorubicin was found in both the tumour periphery and core [35]. The group further developed a heating algorithm using the same rabbit tumour model proving that the use of the binary feedback algorithm results in accurate and homogenous heating within the targeted area [36]. A computational model that simulated the tissue heating with HIFU treatment and the resulting hyperthermia that leads to drug release was developed by Haemmerich. In this model a spatiotemporal multicompartmental pharmacokinetic model simulated the drug release in the blood vessels and its transport into the interstitium as well as cell uptake. Two heating schedules were simulated each lasting 30 min, the first corresponding to hyperthermia, (HT; 43°C) and the second corresponding to hyperthermia followed by a high temperature (50°C) for 20s pulse, (HT+). Using the computational model (validated in rabbit VX2 tumours) the authors found that cellular drug uptake is directly related to hyperthermia duration. HT+ enhanced drug delivery by 40% compared to HT [37]. The study indicates the importance of simulations in the application of drug delivery mechanisms to tumours.

In addition to the progress in the understanding of the physical mechanism of drug delivery from well validated thermosensitive liposomes carrying doxorubicin, researchers further investigated the chemical composition of such liposomes in response to HIFU induced hyperthermia. 

De Smet et al. compared thermosensitive liposomes carrying doxorubicin and ProHance^®^. Two temperature-sensitive systems composed of the following lipids DPPC:MPPC:DPPE-PEG2000 (low temperature-sensitive liposomes; LTSL) and DPPC:HSPC:cholesterol:DPPE-PEG2000 (traditional temperature-sensitive liposomes; TTSL) were investigated for their stability and release profile at 37°C and 42°C in phantoms using MRI 1,2-Dipalmitoyl*-sn-*glycero-3-phosphocholine (DPPC), 1-palmitoyl*-sn-*glycero-3-phosphocholine (MPPC), 1,2-dipalmitoyl*-sn-*glycero-3-phosphoethanolamine-N[methoxy(polyethyleneglycol)-2000] (DPPE-PEG2000), hydrogenated-L-*α*-phosphatidylcholine (HSPC). The LTSL system showed a higher leakage of doxorubicin at 37°C, but a faster release of doxorubicin at 42°C compared to the TTSL system indicating that lipid composition plays an important role on stability and release profile [38]. The authors further investigated the more stable traditional temperature sensitive liposomes carrying doxorubicin and ProHance^®^ *in vivo* in rats bearing 9L gliosarcoma tumours. A clinical MRI-HIFU system was applied in a proof-of-concept study to induce local hyperthermia for 30 min. The local temperature-triggered release of ProHance^®^ was monitored with interleaved *T*_1_ mapping of the tumour. A good correlation between the Δ*R*_1_ (change in longitudinal relaxation rate Δ*R*_1_ = Δ(1/*T*_1_)) and the intratumour doxorubicin and gadolinium concentration was found, implying that the *in vivo* release of doxorubicin from the thermosensitive liposomes can be probed in situ with the longitudinal relaxation time of the coreleased MRI contrast agent (dose painting).

Temperature sensitive liposomes release their encapsulated drugs at the melting phase transition temperature (*T*_*m*_) of the lipid bilayer. At this *T*_*m*_ the lipid membrane changes its structure as it transfers from a gel to the liquid crystalline phase [39]. When the liposomal membranes are in the gel phase they show less permeability to molecules and water compared to the liquid crystalline phase. 

The liposomes' transition to the liquid crystalline phase can be achieved with the incorporation of a lyso-phospholipid such as MSPC (R = −C_17_H_35_). This lipid is also the lipid used in the thermodox^®^ formulation [40]. A potential disadvantage of MSPC containing liposomal formulations is their rapid doxorubicin leakage at 37°C [37]. Tagami et al. prepared temperature sensitive liposomes using nonionic surfactants Brij which are PEG-ylated lysolipids resembling the chemical structures of MSPC and DSPE-PEG(2000). Results indicated that the optimal acyl chain length of the surfactant was between C(16) and C(18) with a saturated carbon chain and a PEG repeating unit ranging between 10 and 100 with a molecule weight above 600 Da. In the panel of surfactants tested, Brij78 was optimal and could be incorporated into the liposomes by the thin film hydration or the postinsertion method with an optimal range of 1 to 8 mol% [41]. The authors continued with *in vivo* experiments in mice bearing mammary carcinoma cells EMT-6, investigating Gd^3+^DTPA (diethylene triamine pentaacetic acid) release with relaxometry. The authors observed a good correlation between relaxation enhancement in the heated tumour and the inhibition of tumour growth at day 21 after treatment [42].

Kono et al. investigated the effect of poly [2-ethoxy(ethoxyethyl)vinyl ether] chains (having a lower critical solution temperatures) and polyamidoamine G3 dendron-based lipids having Gd^3+^ chelate residues into PEGylated liposomes. These designed liposomes exhibited excellent ability to shorten the longitudinal proton relaxation time. When administered intravenously into tumour-bearing mice, accumulated liposomes in tumours increased with time, reaching a constant level 8 h after administration by following *T*_1_-weighted MRI signal intensity in tumours. Liposome size affected the liposome accumulation efficiency in tumours: liposomes of about 100 nm diameter were accumulated more efficiently than those with about 50 nm diameter. Tumour growth was strongly suppressed when liposomes loaded with doxorubicin were administered intravenously into tumour-bearing mice and the tumour was heated mildly at 44°C for 10 min at 8 h after administration [43].

In our group we have investigated the potential of an MRI labelled phospholipid/lysolipid containing liposome to accumulate in tumours and release the drug under conditions of mild hyperthermia induced by FUS.

**Figure 1:**
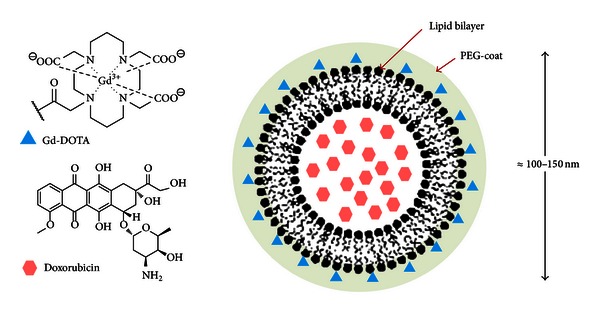
Thermosensitive liposome for real-time monitoring of nanoparticle accumulation in tumours.

We label the liposome nanoparticles with a lipid that consists of a DOTA [1,4,7,10-tetraazacyclododecane-1,4,7,10-tetraacetic acid] headgroup (Figure 1) [44, 45]. Introducing the imaging lipid in the lipid bilayer provides a better and clearer monitoring of liposomal particle kinetics and a better knowledge of the time required for maximum nanoparticles accumulation in tumours (monitored by MRI).

Although most research studies have focused mainly in thermoresponsive liposomes and FUS activation of drug release, there is limited work on the use of polymers (thermoresponsive or not) and their application in FUS triggered drug delivery. The effect of ultrasound on drug release from polymers was studied in 1989 by Kost et al. and indeed the authors found that ultrasound can increase the polymer degradation rate leading to 20 times higher release rate. Interestingly the authors observed that the release rate increased in proportion to the intensity of ultrasound proposing that cavitation appeared to play a significant role [46]. 

### 3.3. Ultrasound and Microbubbles to Increase Drug Permeability in Tissues

Triggered drug delivery using an external physical force provides the required control of drug deposition in certain tissues avoiding exposure of healthy tissues to high (toxic) concentrations. The trigger induced delivery should be acute and the effect induced on nontargeted tissues nondamaging and reversible. Hyperthermia induced by a means like ultrasound can be exploited as an external trigger in drug delivery [3, 47].

Mild hyperthermia can be induced by pulsed FUS that can reduce extreme tissue heating by allowing the tissue to cool down between US exposures [48]. The increase in temperature can be 3–5°C (hyperthermia) despite the high energy deposited in the tissue. Hyperthermia applied in tumours can increase blood flow and enhance vascular permeability. Studies with canine soft tissue sarcoma and human tumour clinical studies have also demonstrated that hyperthermia improves tumour oxygenation and enhances response of such tumours to radiotherapy or chemoradiotherapy. The increased blood flow and vascular permeability caused by temperatures such as 42°C may also improve the delivery of chemotherapy drugs, immunotherapeutic agents and genes to tumour cells [49]. FUS exposures in pulsed mode lower the rates of energy deposition and generate primarily mechanical effects for enhancing tissue permeability to improve local drug delivery. These pulsed exposures can be modified for low-level hyperthermia as an enhancement of drug delivery that would lead to better drug deposition and better therapeutic effect [50]. Mild hyperthermia of 42°C can improve the degree of nanocarrier extravasation as shown by Kong et al. [51]. The reason that this leads to increased extravasation maybe due to downregulation of VE-cadherin that contributes to vascular integrity as it was shown in HUVEC endothelial cells [52]. It is clear that hyperthermia can provide a boost to extravasation and drug deposition in tumours. This should provide an adjuvant effect when nanocarriers are used and accumulate in tumours due to enhanced permeation and retention effect. It would be interesting to investigate the effect of hyperthermia on tumour/tissue drug clearance.

FUS can also induce nonthermal effects on tissues. Acoustic cavitation can be induced using microbubbles exposed to US [53]. Acoustic cavitation can be defined as the growth, oscillation, and collapse of gas containing bubbles under the influence of the varying pressure field of sound waves in a fluid and can have an effect on the permeability of a biological tissue [53–55]. There are two types of acoustic cavitation: noninertial and inertial cavitation. The noninertial (stable) cavitation occurs when bubbles persist for a number of acoustic cycles. In this case the bubble's radius increases and decreases (expands and contracts) according to the applied US frequency. Inertial (transient cavitation) occurs when bubbles grow faster expanding 2- or 3-fold their resonant size, oscillate unstably, and collapse in a single compression half cycle [54]. It has been considered that the primary mechanism to affect the structure of intact cells is inertial cavitation that can induce irreversible damage as well as increase cell membrane permeability [56, 57].

An important application of HIFU and microbubbles lies in the area of altering the permeability of the blood brain barrier (BBB). In a study in 2002, Mesiwala et al. observed that HIFU could alter BBB permeability. HIFU induced reversible, nondestructive, BBB disruption in a targeted area and this opening reversed after 72 h. The authors showed with microscopy that HIFU either entirely preserved brain architecture while opening the BBB, or generated tissue damage in a small volume within the region of BBB opening. Further electron microscopy suggested that HIFU disrupted the BBB by opening capillary endothelial cell tight junctions, a mechanism that was not observed in other methods used to open BBB [58]. 

The effect of FUS on tight junctions' integrity was later confirmed in a study investigating rat brain microvessels after this BBB disruption. The authors used immunoelectron microscopy to identify tight junctional proteins such as occludin, claudin-1, claudin-5, and submembranous ZO-1 after sonication. They found substantial redistribution and loss of occludin, claudin-5 and ZO-1. However, claudin-1 seemed less involved. Monitoring the leakage of horseradish peroxidase (MW 40 KDa) the authors observed that the BBB disruption appears to last up to 4 h after sonication [59]. In a later study the role of caveolin in the mechanism of FUS-BBB enhanced permeation was suggested. In a study investigating caveolae density it was found that caveolae and caveolin-1 were primarily localized in the brain microvascular endothelial cells of all the animals tested (rats) regardless of treatment, and that caveolin-1 expression was the highest in the rats treated with both FUS and microbubbles. The authors concluded that caveolin-1-mediated transcellular transport pathway may cooperate with other transport pathways (e.g., tight junctional disruption) to induce opening of the BBB [60]. 

Hynynen and colleagues investigated the BBB FUS enhanced permeability in rabbits. Rabbit brains were exposed to pulsed focused ultrasound while microbubbles were intravenously administered. The BBB opening was measured by an MRI contrast agent evaluating the local enhancement in the brain. The authors found that low ultrasound powers and pressure amplitudes were found to cause focal enhancement of BBB permeability. Trypan blue injected before animals were sacrificed indicated blue spots in the areas of the sonicated locations [61]. The authors concluded that HIFU disruption of BBB could be used enhancing drug delivery to the brain [62].

McDannold et al. tested the safety of this method by searching for ischemia and apoptosis in areas with BBB disruption induced by pulsed ultrasound in the presence of gas bubbles and by looking for posttreatment effects up to one month after sonication. Pulsed ultrasound exposures (sonications) were performed in the brains of rabbits under monitoring by MRI. BBB disruption was confirmed with contrast-enhanced MR images. Whole brain histologic examination was performed using staining for ischemic neurons and TUNEL staining for apoptosis. Tiny regions of extravasated red blood cells scattered around the sonicated locations, indicated capillaries. Despite these vasculature effects, only a few cells in some of the sonicated areas showed evidence of apoptosis or ischemia. The authors found that ultrasound-induced BBB disruption is possible without inducing substantial vascular damage that would result in ischemic or apoptotic death to neurons [63]. 

The method could find application in the delivery of large therapeutic molecules that do not normally permeate the BBB. Herceptin (trastuzumab), a humanized anti-human epidermal growth factor receptor 2 (HER2/c-erbB2) monoclonal antibody, was delivered locally and noninvasively into the mouse central nervous system through the blood-brain barrier under image guidance by using an MRI-guided focused ultrasound. The amount of herceptin delivered to the target tissue was correlated with the extent of the MRI-monitored barrier opening, making it possible to estimate indirectly the amount of Herceptin delivered. The method could be used to treat breast cancer metastases to the brain [64]. It was further shown that dopamine D(4) receptor-targeting antibody could also be delivered using the same technique in the brain [65, 66].

Delivery of small molecules can also be enhanced with the use of HIFU cavitation disruption of the BBB. Treat et al. demonstrated relatively high concentrations of doxorubicin in the brain with minimal healthy tissue damage effects. The authors observed that doxorubicin accumulation in nontargeted contralateral brain tissue remained significantly lower. MRI signal enhancement in the sonicated region correlated strongly with tissue doxorubicin concentration, suggesting that contrast-enhanced MRI could perhaps indicate drug penetration during image-guided interventions [67].

Konofagou and coworkers assessed the spatial permeability of the BBB-opened region using dynamic contrast-enhanced MRI (DCE-MRI) in mice. The authors processed DCE-MR images using the general kinetic model and the reference region model. Permeability maps were generated and the *K*_trans⁡_ (the transfer rate constant from the intravascular system to the extracellular extravascular space) values were calculated for a predefined volume of interest in the sonicated and the control area for each mouse. The results demonstrated that *K*_trans⁡_ in the BBB-opened region was at least two orders of magnitude higher when compared to the contralateral (control) side [68].

There are several parameters to affect the level of BBB enhanced permeability and the endothelial tight junctions disruption; the pulse sequence comprising short bursts, the spacing between bursts or the rate of infusion of the microbubbles, and the size of microbubbles were found to affect the effect on BBB disruption [69, 70].

The method could be applied for a number of therapeutic applications. The brain-derived neurotrophic factor (BDNF) was delivered to the left hippocampus in mice through the noninvasively disrupted blood-brain barrier (BBB) using focused ultrasound. The BDNF bioactivity was found to be preserved following delivery as assessed quantitatively by immunohistochemical detection of the pTrkB receptor and activated pAkt, pMAPK, and pCREB in the hippocampal neurons. It was shown that BDNF delivered this way induced signalling effects in a highly localized region in the brain [71]. 

However it is the area of targeting brain tumours that have attracted most interest in the FUS disrupted BBB [72]. Mei and colleagues investigated the effects of targeted and reversible disruption of the blood-brain barrier by MRI-guided focused ultrasound and delivery of methotrexate to the rabbit brain. The authors recorded that the methotrexate concentration in the sonicated group was notably higher than that in both the control group (intravenous administration) and the internal carotid artery administered group. They observed a greater than 10-fold increase in the drug level compared to internal carotid administration without FUS [73].

Liu et al. investigated the delivery of 1,3-bis(2-chloroethyl)-1-nitrosourea (BCNU) to glioblastomas in rats with induced tumours with the help of FUS. The authors found that FUS significantly enhanced the penetration of BCNU through the BBB in normal and tumour-implanted brains without causing bleeding. Surprisingly, treatment of tumour-implanted rats with focused ultrasound alone had no beneficial effect on tumour progression. However, treatment with focused ultrasound before BCNU administration controlled tumour progression and improved animal survival relative to untreated controls [74].

Liu and colleagues recently assessed FUS-mediated delivery of an iron oxide magnetic nanoparticle (MNPs) conjugated to an antineoplastic agent, epirubicin. They used MNPs because of the favourable MR imaging characteristics, which could facilitate imaging. They demonstrated a substantial accumulation of MNPs, as well as epirubicin, up to 15 times the therapeutic range in the brain when delivered with FUS. They further showed decreased tumour progression in animals with brain tumours that received MNP with epirubicin via FUS [75].

Receptors targeting liposomal nanocarriers have been combined with MRgFUS to treat brain tumours. In a recently presented study it was shown that pulsed HIFU and human atherosclerotic plaque-specific peptide-1- (AP-1-) conjugated liposomes containing doxorubicin (AP-1 Lipo-Dox) acted synergistically in an experimental brain tumour model. Prior to each sonication, AP-1 Lipo-Dox or unconjugated Lipo-Dox were administered intravenously, and the concentration in the brain was quantified. Drug injection with sonication increased the tumour-to-normal brain doxorubicin ratio of the target tumours by about twofold compared with the control tumours. Moreover, the tumour-to-normal brain ratio was the highest after the injection of AP-1 Lipo-Dox with sonication. The results of this study indicate that combining targeting strategies can substantially enhance delivery of chemotherapy in the brain [76]. In a separate study the authors investigated the pharmacokinetics of ^111^I-labeled AP1-Lipo-dox using microSPECT. The authors confirmed that sonication increased liposomal doxorubicin concentrations in tumour areas (murine glioblastoma) and that molecular targeting acts synergistically with FUS [77]. 

Targeted gene transfer into central nervous system was investigated using MRI-guided focused ultrasound-induced blood-brain barrier disruption. The results of this study showed that MRI-guided FUS achieved plasmid DNA transfer across the opened BBB furthermore plasmid ware internalized into the neurons presenting heterogeneous distribution and numerous transparent vesicles were observed in the cytoplasm of the neurons in the sonicated region, suggesting vesicle-mediated endocytosis. BDNF (and BDNF-EGFP) expressions were markedly enhanced by the combination of ultrasound and pBDNF-EGFP-loaded microbubbles about 20-fold than that of the control group. The method by using MRI-guided FUS to induce the local BBB disruption could accomplish effective targeted exogenous gene transfer in the CNS. In this study the microbubbles were used as the plasmid carrier. The investigators conjugated plasmid onto the surface of microbubbles and they coated these carriers using polymers in a layer by layer technique [78].

An exciting application is the delivery of therapeutic stem cells to the brain using FUS to potentially treat neurodegenerative diseases, traumatic brain injury, and stroke. MRI guidance was used to target the ultrasound beam thereby delivering iron-labeled, green fluorescent protein (GFP) expressing neural stem cells specifically to the striatum and the hippocampus of the rat brain. Immunohistochemical analysis confirmed the presence of GFP-positive cells in the targeted brain regions suggesting that MRIgFUS may be an effective alternative to invasive intracranial surgery for stem cell transplantation [79].

Although a very efficient approach, the use of microbubbles to enhance drug permeation through tissues, it may require significant safety consideration. In a key study in 2005 Prentice et al. presented clearly in a well-designed experimental setup that there are important interactions between individual cells and violently cavitating microbubbles leading to large pores in the cell membrane (sonoporation) [80]. These effects on cell membrane will need to be thoroughly investigated at microscopical and molecular level to design efficient and safe FUS regimes. 

### 3.4. Drug Delivery Dosage Forms and FUS Future Perspective

During the last few years there has been an expansion in research in MRgFUS drug delivery. The main dosage forms tested in MRgFUS drug delivery strategy are the thermosensitive liposomes and the lipid based microbubbles that can be conjugated with drugs or other liposomes on their surface [78, 81].

There is limited research in the area of using other responsive materials or nanocarriers. Rapoport discussed recently the potential of using micelles and FUS [82] for enhanced tissue permeation. Micelles are nanosized carriers able to carry hydrophobic drugs; their combination with FUS could substantially enhance their delivery in tissues. Kostarelos and colleagues suggested the incorporation of thermosensitive peptides onto liposome bilayers to enhance thermoresponsiveness [83], and the group of Lammers designed polymer-based microbubbles for ultrasound drug release [84].

It is clear that already established delivery systems such as different structurally nanocarriers have not been investigated in combination with image guided FUS. It would be interesting to see the effect of FUS on the enhanced permeability of micelles, polymers (dendrimers cyclodextrins), or metal nanoparticles (gold-iron) to tissues. Thermosensitive materials have been hardly explored in this field. Polymers or proteins that respond to small change of temperature could form suitable image guided FUS triggered platforms. 

The effects of FUS in biological tissues with or without carriers will require a more thorough investigation to understand the short- and long-term effects of ultrasound in the body and the complex environments such as tumours, blood vessels, and bone. The mechanism of FUS induced hyperthermia and/or the FUS tissue permeability increase is not well understood at cellular and molecular levels. There is limited knowledge on the effects of FUS on genomic DNA and if certain proteins are overexpressed after FUS treatment. 

In addition to the above, the frequency of FUS drug delivery treatments (or dosing) and the long-term effects in the body will have to be investigated in preclinical studies in order to design a FUS drug treatment regime. 

An imaging modality will have to be used for accurate image guided FUS therapy. In the case of MRI clinically approved contrast enhancing agents will have to be added to the delivery system to monitor carriers' distribution in the treatment area as well as efficient and rapid release. 

Considering the approval in clinical applications, such treatments will require the control of several factors such as drug and drug carrier, MRI contrast enhancing agents, and MRgFUS parameters, and this could mean several regulatory hurdles. However, the fact that most of the components (FUS, liposomes) have been tested in clinical trials is encouraging for such approach to move forward. 

Most of the current strategies to increase tumour specificity of nanocarriers include the use of tumour biomarkers for either targeting (receptors) or for triggered release (internal stimuli; pH proteases) and/or the use of external stimuli such as light and ultrasound. Biomarkers and internal stimuli may vary in different tumours indicating that such nanocarriers for cancer treatments should be “individualised.” External stimuli can be used independent the tumours characteristics and therefore guarantee a more uniform effect. FUS can be used as an external stimulus to activate drug delivery in tissues. It also shows the significant advantages of being noninvasive as well as controlled and focused. 

Overall MRgFUS drug delivery is a novel and valuable tool to increase drug targeting and tissue specific drug delivery. It is expected that future studies will prove the clinical efficacy of MRgFUS drug delivery applications.
